# Mediating effects of meaning in life on the relationship between family care, depression, and quality of life in Chinese older adults

**DOI:** 10.3389/fpubh.2023.1079593

**Published:** 2023-04-03

**Authors:** Jing-Jing Zhou, Yu Zhang, Qing-Zhuo Ren, Ting Li, Gui-Ding Lin, Min-Yi Liao, Shao-Hua Chen, Pei Tong, Yu-Lin Gao

**Affiliations:** ^1^School of Nursing, Southern Medical University, Guangzhou, China; ^2^School of Public Health and Management, Guangzhou University of Chinese Medicine, Guangzhou, China; ^3^School of Health Management, Southern Medical University, Guangzhou, China; ^4^Guangzhou First People’s Hospital, Guangzhou, China; ^5^Liurong Community Health Service Center in Yuexiu District, Guangzhou, China; ^6^Jingxi Community Health Service Center in Baiyun District, Guangzhou, China

**Keywords:** depression, sources of meaning, meaning in life, older adults, quality of life, successful aging

## Abstract

**Background:**

The study explored sources of meaning in older adults and the action path among family care, meaning in life, quality of life, and depression.

**Materials and methods:**

We investigated 627 older adults using the Sources of Meaning in Life Scale for the Elderly (SMSE), the Family Care Index (APGAR), the Center for Epidemiological Studies Depression Scale-10 (CES-D-10), and the EuroqOL-5 Dimensions (EQ-5D).

**Results:**

Scores categorized 454 older adults with good family function, 99 with moderate, and 47 with severe family dysfunction; 110 older adults had depression. The structural equation model showed that family care affected the quality of life and depression by influencing meaning, and depression had a significant negative effect on the quality of life (*P* < 0.05). The model was a good fit for the data (χ^2^/df = 3.300, SRMR = 0.0291, GFI = 0.975, IFI = 0.971, TLI = 0.952, CFI = 0.971, RMSEA = 0.062).

**Conclusion:**

Meaning in life is an intermediary factor that affects depression and quality of life in older adults. Family care had a significant positive impact on SMSE and a negative influence on depression. The SMSE effectively clarifies the sources of meaning in life and can be used to improve meaning and promote mental health in older adults.

## 1. Introduction

The Seventh National Census (1) showed that China’s aging population reached 264 million in 2020, accounting for 18.7% of the country’s total population. The aging process is accelerating, and older adults’ physical and mental health requires attention. Older adults are prone to negative emotions, such as loneliness, depression, and even suicide (2). A systematic review and meta-analysis in 2022 showed that the global prevalence of depression in older adults was 28.4% (3) and accounted for 36% of suicides in China (4). More than 100,000 older adults die of suicide every year, and most were experiencing depression (5) and a decreased quality of life (QoL) because of weakness or disease in addition to depression. A 2010 study found that depression and health satisfaction mediated the indirect effect of the environment on QoL (6).

Social support is a heterogeneous concept, with multiple pathways by which it may influence mental and physical health. Social support promotes health by influencing both psychological and behavioral processes. For instance, relevant psychological pathways include stress appraisals, and behavioral pathways include health behavior change (7). Careful attention to these issues is needed when developing effective social support interventions. As a form of social support, family care also affects depression and quality of life. Frankl asserted that family care is a source of meaning in life (MIL). This unique ability of humans to perceive meaning can be positively stimulated to help overcome suffering. Logotherapy was the first therapy designed for this purpose (8). Logotherapy has been adopted by mental health professionals across various theoretical orientations and involves influencing the construction of meaning in human health and behavior. There is growing evidence supporting the relationship between a sense of meaning and both psychological and physical wellbeing. Logotherapy awakens the MIL and guides people to respond to their circumstances, thus reducing depression and improving the quality of life (9). This study explored the relationship among four variables based on the theoretical framework of social support theory and logotherapy.

Meaning in life studies in older adults began in the 1980s and focused on the sense of meaning and its influence (10). Research showed that older adults’ MIL was influenced by many factors, including age, gender, cultural background, physical health, depression, loneliness, subjective wellbeing, experience, living environment, economic level, and interpersonal relationships (10, 11). MIL decreases with increasing age, and older adults with poor physical health are more likely to experience a sense of meaninglessness (11). Moreover, MIL can significantly affect physical and mental health and interpersonal intimacy (5). From the perspective of positive psychology, MIL helps to relieve loneliness, depression, and other negative emotions and improves subjective wellbeing and quality of life (12, 13). MIL also plays a role in promoting healthy aging and reducing the risk of death (10).

Researchers have not yet developed a unified MIL definition; research has focused on breadth and depth perspectives. In breadth, MIL is a multidimensional concept developed from various sources (10, 14). In previous studies, we developed a tool based on the breadth and depth perspectives of MIL regarding sources and levels of meaning for older adults (15). Studies showed that finding an individual’s sources of meaning can help boost MIL. Family care is also an important source of MIL for older adults and affects their sense of MIL. Family and social support can reduce the incidence of depression and alleviate depressive symptoms (16, 17). A randomized controlled trial on older patients with depression showed that reconstruction of meaning can reduce depression (18). Yang (19) indicated that older women mostly acquired MIL from family and belonging. Rong (20) concluded that the most important sources of meaning were material things and security, followed by family suffering, physical health, leisure, and life experience. Sources of meaning can actively promote MIL and shift an individual’s life attitude from negative to positive (21). Therefore, by integrating family support into older adults’ daily life and developing initiatives that promote healthy surroundings, such as social connectedness, co-residential living, and special care for those who are physically disabled, we can protect older adults against late-life depression (22).

Meaning in life is important for the quality of life; patients with a positive sense of MIL feel better, have more energy, and thus have a higher quality of life. MIL involves creating a connection between our inner depths and the outer world; finding a dream and all the hidden potentials and fighting to bring them out, which reflects the quality of life as life self-realization (23).

Successful aging refers to older adults’ abilities to manage the physiological, psychological, and social challenges of aging, where they achieve balance or an optimal state between self and environment (24). Mu (25) believed that the key to successful aging involves using the positive energy of “gain,” such as MIL, family care, and social support to balance the negative energy of “loss,” such as aging, declining health status, and an empty nest. “Gain” can play a protective role and help older adults succeed in aging. However, this view is still in the theoretical research stage and lacks empirical research in China. No relevant literature has analyzed pathways among the meaning of life, family care, depression, and quality of life.

Therefore, this study focuses on exploring older adults’ sources of meaning and the meaning obtained from each source and explores the MIL mediating effects on relationships between family care, depression, and quality of life in Chinese older adults. Based on the theoretical demonstration of successful aging, we hypothesized that family care promotes MIL in older adults, and thereby improves their physical and mental health.

## 2. Materials and methods

### 2.1. Procedures and participants

The Medical Ethics Committee of the Southern Medical University of Nanfang Hospital in China approved the study, and survey administration was conducted according to relevant guidelines and regulations. Participants provided voluntarily written informed consent and did not interact. Inclusion criteria were permanent residents of Guangzhou and aged 60 years or older. Exclusion criteria were mental illness diagnosis, a definite diagnosis of severe physical illness (including advanced cancer, heart, liver, and kidney failure), and declining to participate. The sample size was calculated according to the formula provided by Wu (26):

$$n={\left(U_{\frac{\alpha }{2}}\times \frac{\sigma }{\delta }\right)}^{2}$$

Forty-seven older adults from the Community Health Service Center A were selected as participants in the preliminary investigation. The standard deviation σ was 40.418, tolerance error δ was 4 in the pre-survey, and the significance level α was 0.05, and considering a 10% sample loss rate, the sample size was estimated to be 433.

Data collection took place from June to September 2018. We administered a cross-sectional survey to 627 older adults from six sites using a convenience sampling method. Of these, 601 (95.9%) returned questionnaires were valid, including those from the Community Health Service Center A (139, 23.1%), Community Health Service Center B (95, 15.8%), Hospital A (100, 16.7%), Hospital B (86, 14.3%), Nursing Home A (44, 7.3%), and Nursing Home B (137, 22.8%). There were 257 (42.8%) male and 344 (57.2%) female participants; 54 (9.0%) had religious beliefs and 543 (90.3%) had no religious beliefs; 164 (27.3%) lived alone and 435 (72.4%) lived with others; 374 (62.2%) had a spouse and 227 (37.8%) had no spouse or their spouse had died; 303 (50.4%) were local and 296 (49.3%) were non-local older adults; the average age was 77.04 ± 9.336 years, ranging from 60 to 100 years. Individuals’ data were anonymous and collected without identifiable personal information.

### 2.2. Instruments

A sociodemographic questionnaire gathered information on participants’ age [a continuous variable, dichotomized according to the division of older adults in China into young-old (60–80 years) and old-old (81–100 years)], gender, religious or not, living alone or not, educational attainment (no schooling, primary school, technical secondary school/junior high school, senior high school, junior college, and bachelor or above), marital status (single, divorced, widowed, and married), Guangzhou native or not, previous occupation, personal monthly income, self-perceived economic status, physical health status, degree of self-care, life satisfaction, personality, number of chronic diseases, disease burden, anxiety, loneliness, and view of life.

The Sources of Meaning in Life Scale for the Elderly (SMSE) (15) is based on the breadth and depth perspectives of MIL to identify the sources and level of meaning of older adults and is suitable for Chinese older adults. The questionnaire includes 28 items under six dimensions (family, personal development, social support, sense of value, leisure activities, and life security), and uses a 7-point Likert scale to measure MIL intensity from each source, ranging from 1 (absolutely not meaningful) to 7 (very meaningful). The total score in our sample ranged from 28 to 196 points, where higher scores reflected a stronger sense of MIL. Cronbach’s alpha was 0.924, and the six dimensions ranged from 0.727 to 0.870.

The Family Care Index Scale (APGAR) (27) includes five items that measure adaptation, partnership, growth, affection, and resolve, scored from 0 to 2 points; the total score is 0–10 points, where higher scores reflect a higher family care index. Scores from 7 to 10 indicate good family function 4–6 indicate moderate family dysfunction; and 0–3 indicate severe family dysfunction. Moderate and severe family dysfunction reflects low family care, and good family function reflect high family care. Cronbach’s alpha in this study was 0.890.

The Center for Epidemiological Studies Depression Scale-10 Item Version (CES-D-10) (28) includes 10 items scored from 0 to 3, representing few or none, some days, half the time, and often or almost every day, respectively. The total score ranges from 0 to 30, where higher scores indicate more serious depression. A score of 10 or above indicates a tendency toward depression. Cronbach’s alpha in this study was 0.794.

The EuroQol-5 Dimensions (EQ-5D) (29) include the EQ-5D health description system and EQ visual analog scale (EQ-VAS). The health description system includes five dimensions: self-care ability, daily activity capacity, pain or discomfort, anxiety, and depression. Each dimension is scored as no difficulty, some difficulty, and extreme difficulty. After conversion, the score ranges from −0.59 to 1.00. EQ-VAS is a vertical scale that expresses self-conscious physical health status, from 0 points (the worst health status in mind) to 100 points (the best health status in mind). The scale is widely used in China. Cronbach’s alpha in this study was 0.822.

### 2.3. Statistical analysis

We used Epidata 3.1 to double-enter the data, and IBM SPSS Statistics 22.0 and AMOS Statistics 24.0 to analyze the data. Descriptive statistics were used to analyze general demographic variables. Median and quartile spacing M (*P*_25_, *P*_75_) were used to describe SMSE, APGAR, depression, and quality of life scores because the data did not conform to a normal distribution. Cronbach’s alpha coefficients were used to test each scale’s internal consistency reliability. We used Spearman correlation analysis to measure relationships among SMSE, APGAR, CES-D-10, and quality of life. The Mann–Whitney U-test and the Kruskal–Wallis test were used to analyzing SMSE differences by demographics. We used structural equation modeling to analyze the relationships among MIL, family care, quality of life, and depression. *P*-values less than 0.05 were considered statistically significant.

## 3. Results

### 3.1. Participant characteristics

The median SMSE score was 166.00 (147.00, 179.00), ranging from 47 to 196, which was a high level. The repeated measure ANOVA showed that the family harmony and art hobby items had the highest and lowest scores, respectively (*F* = 330.429, *P* < 0.01). Dimension scores ranked from highest to lowest were life security, family, sense of value, social support, leisure activity, and personal development (*F* = 125.665, *P* < 0.01), as shown in Table 1. The median APGAR score was 9.00 (7.00, 10.00), and ranged from 0 to 10; 454 (75.5%) participants had a good family function, 99 (16.5%) had moderate family dysfunction, and 47 (7.8%) had severe family dysfunction. Older adults with a low family care index accounted for 24.3% of the sample. The median EQ-5D index was 0.796 (0.691–1.000), ranging from −0.59 to 1. The median VAS was 70 (60, 80), ranging from 0 to 100. The median CES-D-10 score was 4.00 (1.00, 8.00), ranging from 0 to 27; 110 participants were positive for depression (18.3%).

**Table 1:** Score of the Sources of Meaning in Life Scale for the Elderly (*N* = 601).

	Scores *M* (*P*_25_, *P*_75_)	Score ranges	Item average score *M* (*P*_25_, *P*_75_)
Total score	166.00 (147.00, 179.00)	47–196	5.93 (5.25, 6.39)
Life security	26.00 (24.00, 28.00)	12–28	6.50 (6.00, 7.00)
Family	25.00 (22.00, 28.00)	4–28	6.25 (5.50, 7.00)
Sense of value	43.00 (38.00, 47.00)	12–49	6.14 (5.43, 6.71)
Social support	24.00 (19.00, 26.00)	4–28	6.00 (4.75, 6.50)
Leisure activities	29.00 (24.00, 32.00)	5–35	5.80 (4.80, 6.40)
Personal development	22.00 (17.00, 26.00)	4–28	5.50 (4.25, 6.40)

### 3.2. Meaning in life’s effect on the association between family and depression or quality of life

Influencing SMSE factors are shown in Table 2.

**Table 2:** Demographic data and scores of SMSE in different groups.

Items		*n* (%)	Scores or mean rank	*Z*	*P*
Family care	High	454 (75.5)	169.00 (152.00, 182.00)^a^	6.565	*
	Low	146 (24.3)	152.00 (133.00, 170.00)^a^		
Depression or not	Yes	110 (18.3)	152.00 (127.00, 170.00)^a^	6.166	*
	No	491 (81.7)	168.00 (151.00, 180.00)^a^		
Age group	>80	255 (42.4)	172.00 (156.00, 184.00)^a^	4.995	*
	≤80	346 (57.6)	161.00 (143.00, 176.00)^a^		
Native or not	Yes	303 (50.4)	164.00 (144.00, 178.00)^a^	2.352	0.019
	No	296 (49.3)	168.00 (149.25, 182.75)^a^		
Religious or not	Yes	54 (9.0)	172.50 (159.00, 181.25)^a^	2.131	0.033
	No	543 (90.3)	165.00 (147.00, 178.00)^a^		
Gender	Male	257 (42.8)	164.00 (143.50, 178.00)^a^	1.538	0.124
	Female	344 (57.2)	168.00 (149.00, 180.00)^a^		
Living alone or not	Yes	164 (27.3)	165.50 (143.00, 178.75)^a^	0.684	0.494
	No	435 (72.4)	167.00 (148.00, 179.00)^a^		
Have a spouse or not	Yes	374 (62.2)	167.00 (147.00, 178.00)^a^	0.315	0.753
	No	227 (37.8)	166.00 (148.00, 179.00)^a^		
Survey sites	Community A	139 (23.1)	266.23^b^	41.088	*
	Community B	95 (15.8)	289.18^b^		
	Hospital A	100 (16.6)	330.64^b^		
	Hospital B	86 (14.3)	257.03^b^		
	Nursing home A	44 (7.3)	403.00^b^		
	Nursing home B	137 (22.8)	338.48^b^		
Family functions	Good	454 (75.5)	327.42^b^	45.894	*
	Moderate dysfunction	99 (16.5)	226.13^b^		
	Severe dysfunction	47 (7.8)	197.14^b^		
Loneliness	Seldom	473 (78.7)	320.03^b^	29.184	*
	A few days	59 (9.8)	241.92^b^		
	Half the time	26 (4.3)	259.52^b^		
	Always	43 (7.2)	197.83^b^		
Anxiety	Always	63 (10.5)	339.97^b^	45.328	*
	Occasionally	201 (33.4)	266.70^b^		
	Never	337 (56.1)	201.96^b^		
Views on life	Very imperfect	8 (1.3)	21.69^b^	154.135	*
	A little imperfect	42 (7.0)	132.27^b^		
	Ordinary	152 (25.3)	229.20^b^		
	Perfect	272 (45.3)	322.43^b^		
	Very perfect	124 (20.6)	418.52^b^		
Disease burden^#^	No	335 (55.7)	279.07^b^	28.055	*
	A little	96 (16.0)	231.90^b^		
	Moderate	36 (6.0)	193.57^b^		
	Heavy	36 (6.0)	185.31^b^		
	Very heavy	8 (1.3)	223.12^b^		
Medical insurance	Self-paying	24 (4.0)	231.42^b^	27.748	*
	Medical insurance	353 (58.7)	275.39^b^		
	Government insurance	223 (37.1)	347.68^b^		
Life satisfaction	Very dissatisfied	5 (0.8)	127.30^b^	80.018	*
	Dissatisfied	27 (4.5)	192.80^b^		
	Moderate	83 (13.8)	237.48^b^		
	Satisfied	314 (52.2)	278.80^b^		
	Very satisfied	171 (28.5)	393.00^b^		
Personality	Very pessimistic	3 (0.5)	86.00^b^	85.693	*
	Pessimistic	21 (3.5)	158.62^b^		
	Ordinary	185 (30.8)	235.84^b^		
	Optimistic	276 (45.9)	314.68^b^		
	Very optimistic	115 (19.1)	401.99^b^		
Degree of self-care	Self-care	476 (79.2)	304.38^b^	2.235	0.327
	Partly self-care	118 (19.6)	292.56^b^		
	Cannot self-care	7 (1.2)	213.57^b^		
Physical health	Very bad	29 (4.8)	206.48^b^	25.640	*
	Bad	127 (21.1)	266.93^b^		
	Moderate	252 (41.9)	298.25^b^		
	Good	176 (29.3)	336.10^b^		
	Very good	17 (2.8)	394.18^b^		
Economic status	Comfortable income	177 (29.5)	362.68^b^	58.582	*
	Roughly enough	223 (37.1)	310.12^b^		
	Moderate	154 (25.6)	251.58^b^		
	A little difficult	31 (5.2)	176.65^b^		
	Very difficult	15 (2.5)	181.93^b^		
Monthly income	≤1,000	49 (8.2)	178.01^b^	49.309	*
	1,001–3,000	76 (12.6)	248.84^b^		
	3,001–5,000	231 (38.4)	294.08^b^		
	≥5,000	244 (40.6)	347.27^b^		
Previous occupation	Manual labor	177 (29.5)	240.30^b^	39.473	*
	Mental labor	391 (65.1)	332.38^b^		
	No	31 (5.2)	232.53^b^		
Educational attainment	No schooling	35 (5.8)	267.53^b^	21.337	*
	Primary school	107 (17.8)	268.75^b^		
	Technical secondary school/ junior high school	204 (33.9)	283.68^b^		
	Senior high school	98 (16.3)	302.54^b^		
	Junior college	83 (13.8)	362.86^b^		
	Bachelor or above	74 (12.3)	340.07^b^		

^#^Older adults with chronic diseases were surveyed; ^a^Score; ^b^Mean rank; *P < 0.01.

Spearman correlation analysis showed that SMSE was negatively correlated with CES-D-10 (*r* = −0.316, *P* < 0.01), and positively correlated with APGAR (*r* = 0.322), EQ-VAS (*r* = 0.266), age (*r* = 0.216), number of children (*r* = 0.090), and political party belief (*r* = 0.409, *P* < 0.05). In the non-parametric test and correlation analysis, variables that showed a significant impact on SMSE were the independent variables and the SMSE was a dependent variable for multiple stepwise linear regression analysis. Depression score, monthly income (≤1,000, 1,001–3,000, 3,001–5,000, and ≥ 5,000 were assigned 1–4, respectively), religious belief (no and yes were assigned 0 and 1, respectively), personality (very pessimistic, pessimistic, general, optimistic, and very optimistic were assigned 1–4, respectively), and the APGAR were included in the regression equation (*R*^2^ = 0.325, adjusted *R*^2^ = 0.318) (Refer to Table 3).

**Table 3:** Multivariate stepwise regression analysis of scores of the SMSE (*N* = 601).

Items	B	E	*β*	*t*	*P*
Constant	112.463	6.618		16.992	*
Depression	−1.176	0.196	−0.257	−6.002	*
Monthly income	6.518	1.066	0.221	5.775	*
Personality	5.958	1.289	0.194	4.622	*
Family care	1.509	0.380	0.160	3.968	*
Religious or not	12.762	3.301	0.145	3.867	*

*P < 0.01.

We used AMOS 24.0 to test the mediating effect of MIL on family care and depression according to mediating steps (30). First, taking the family care index as an independent variable and depression as a dependent variable, the model’s standardized path coefficient was −0.322 (*P* < 0.01), indicating that family care had a significant negative predictive effect on depression. With family care index and SMSE as independent variables and depression as a dependent variable, MIL had a significant negative predictive effect on depression (*β* = −0.347, *P* < 0.01), family care had a significant positive effect on predicting MIL (*β* = 0.284, *P* < 0.01), and family care had a significant negative effect on predicting depression (*β* = −0.224, *P* < 0.01). This suggests that MIL has a partial mediating effect between family care and depression; the proportion of mediating effect in the total effect was 30.6%. Model fitting indices were as follows: χ^2^/df = 3.769, SRMR = 0.0276, GFI = 0.977, IFI = 0.973, TLI = 0.952, CFI = 0.972, and RMSEA = 0.068, as shown in Table 4.

**Table 4:** Results of path analysis (*N* = 601).

Step	Path	Unstandardized coefficients	Standardized coefficients	S.E.	C.R.	*P*
1	Family care→Depression	−0.443	−0.224	0.077	−5.784	*
	Family care→Meaning in life	0.233	0.284	0.039	5.963	*
	Meaning in life→Depression	−0.837	−0.347	0.116	−7.191	*
2	Family care→Quality of life	0.162	0.025	0.272	0.594	0.553
	Family care→Meaning in life	0.233	0.284	0.039	5.930	*
	Meaning in life→Quality of life	2.034	0.257	0.386	5.269	*
3	Family care→Depression	−0.444	−0.224	0.077	−5.798	*
	Family care→Meaning in life	0.232	0.283	0.039	5.941	*
	Meaning in life→Quality of life	0.728	0.092	0.351	2.072	0.038
	Meaning in life→Depression	−0.839	−0.347	0.117	−7.192	*
	Depression→Quality of life	−1.348	−0.410	0.134	−10.074	*

*P < 0.01.

Similarly, we successively tested the mediating effect of MIL on family care and quality of life. The standardized path coefficient from family care to quality of life was 0.098 (*P* < 0.05), indicating that family care had a significant positive predictive effect on the quality of life. Next, family care and MIL were independent variables and quality of life was a dependent variable, and results showed that MIL had a significant positive predictive effect on the quality of life (*β* = 0.257, *P* < 0.01) and family care had a significant positive predictive effect on MIL (*β* = 0.284, *P* < 0.01), but family care did not predict the quality of life (*β* = 0.025, *P* > 0.05), indicating that MIL has a complete mediating effect between family care and quality of life. The model fitting indices were as follows: χ^2^/df = 3.855, SRMR = 0.0295, GFI = 0.974, IFI = 0.968, TLI = 0.947, CFI = 0.968, and RMSEA = 0.069, as shown in Table 4.

**Figure 1:**
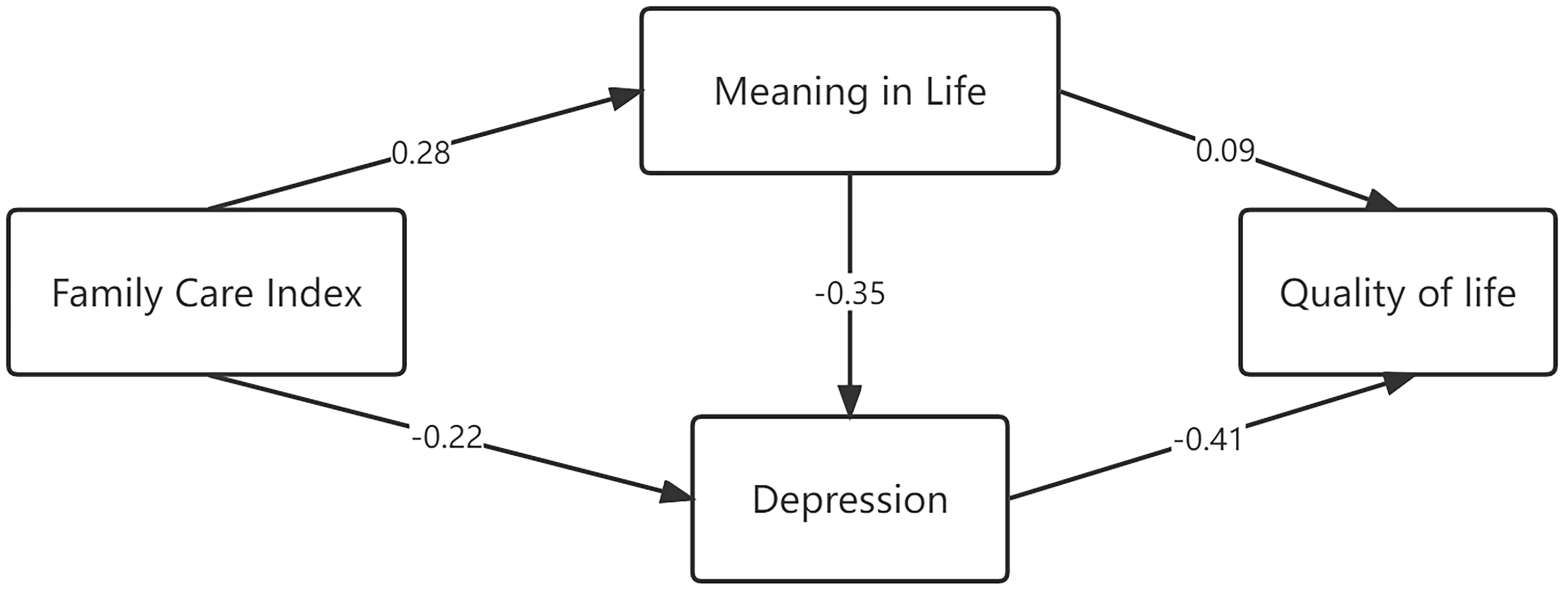
Pathways among meaning in life, family care index, depression, and quality of life.

Then, we explored the relationship among family care, SMSE, quality of life, and depression. Results showed that family care had a significant positive impact on SMSE (*β* = 0.283, *P* < 0.01), family care had a significant negative influence on depression (*β* = −0.224, *P* < 0.01), SMSE had a significant negative effect on depression (*β* = −0.347, *P* < 0.01), SMSE had a significant positive influence on the quality of life (*β* = 0.092, *P* = 0.038), and depression had a significant negative impact on the quality of life (*β* = −0.410, *P* < 0.01). The model fitting indices were as follows: χ^2^/df = 3.300, SRMR = 0.0291, GFI = 0.975, IFI = 0.971, TLI = 0.952, CFI = 0.971, and RMSEA = 0.062, as shown in Table 4 and Figure 1.

## 4. Discussion

Participants’ high meaning score (166 [147, 196]) may be related to relatively high living security. Some participants were retired cadres, and some were from high-end or mid-to-high-end nursing homes. The dimension average scores’ high to low rankings (life security, family, sense of value, social support, leisure activity, and personal development, respectively) are consistent with Maslow’s hierarchy of needs (31). Participants’ meaning came first from the satisfaction of physiological needs and security safeguards, then they gradually sought higher meaning, such as love, belonging, and self-realization.

Of the survey respondents, 110 (18.3%) were positive for depression, which is higher than the reported depression rate in the Guangzhou community (10.8%) (32) and lower than that reported by Lu et al.’s (33) study of in-home care for older adults in the Yuexiu District, Guangzhou (23.5%). Depression has more negative effects on older adults, their families, and even society; it affects their health and cognitive function as an intermediary variable, aggravating the burden of family and society, and even leading to suicide and wounding behavior (5). Thus, depression in older adults needs attention.

Sources of Meaning in Life Scale for the Elderly scores were negatively correlated with depression and had a negative predictive effect on depression, indicating that older adults with a strong sense of meaning have lower depression levels, which is consistent with previous research (2, 34) showing that older adults with a strong sense of meaning have better physical and mental health, and that lack of MIL may lead to existential emptiness, which affects physical and mental health (2, 34). Moreover, mental health was closely related to physical health, interpersonal relationships, quality of life, life satisfaction, and life expectancy, which affect older adults’ successful aging (11). Positive psychology asserts that SMSE can help relieve loneliness, depression, and other negative emotions, improve subjective wellbeing, and promote successful aging (12, 32).

The family care index was positively correlated with MIL, which is consistent with previous results (27). MIL is related to the environment in which good interpersonal relationships are an important source of meaning for older adults (35). Family care is the most important source of meaning; therefore, relationships with family members and family care can affect older adults’ MIL (21, 36).

Family care was negatively correlated with depression. Depression rates were higher in older adults who live alone, have no spouse, and have low family care than in those who live with others and have spouses. Older adults who live alone have less communication with their families and lack family care. In addition, the death of a spouse can lead to negative emotions such as sadness and depression (37). The age gap and lack of communication with family members may also result in low family care for older adults. These results suggested that high levels of family care provide a protective factor for older adults’ mental health and reduce the incidence of depression. Many studies also showed that higher family care levels and social support reduce depression incidence and that peer support and social interaction can also alleviate depressive symptoms in older adults (16). Therefore, improving family care may reduce older adults’ depression incidence.

Sources of Meaning in Life Scale for the Elderly had a significant positive influence on the quality of life, which is consistent with previous results (38). A study of MIL and quality of life after recovery showed that MIL can promote motivation to exercise, which can lead to recovery and improve the quality of life. A rehabilitation study showed that logotherapy is the basis for many psychotherapies (22). This study’s findings may reflect the sense of MIL that can give people a positive attitude toward life, a better social network, and thus a better quality of life.

As a mediating variable, MIL reduces the impact of low family support on depression in older adults, thus promoting their mental health and quality of life. The direct effect of MIL on quality of life was weak; quality of life was improved by reducing depression, which is in line with the theory of successful aging, where the “gain,” such as family care and MIL, can balance the “loss,” such as depression and low quality of life (25, 39). Research showed that MIL was an important factor affecting the quality of life in older adults left behind in rural areas (40). In addition, older adults with a strong sense of MIL tend to have more sources of meaning and a higher sense of value and happiness (31). In this study, older adults with a higher level of family care also had a higher MIL from family and tended to be more optimistic and open-minded, resulting in a lower incidence of depression and a better quality of life. Therefore, family members should give more care to older adults. However, in the 21st century, when young people’s work pressure is increasing sharply, some older adults may not receive good family care. Therefore, we need to stimulate other senses of MIL to stimulate older adults’ interest in creating value, or experiencing the meaning of suffering. In addition, the SMSE can help primary health workers quickly identify sources of meaning for older adults, improve their MIL, promote their mental health, improve their quality of life, and help them achieve successful aging.

We found that more optimistic older adults with higher monthly incomes had higher SMSE scores, which is consistent with other studies (35). In this study, the high monthly income provided certain material and security comforts to older adults, which can affect their MIL to a certain extent. Optimistic and open-minded older adults face hardships and calmly accept reality, have interests and hobbies, and are more willing to communicate with others, further boosting their MIL. SMSE scores of participants with religious beliefs were higher than those of non-religious participants, because they regularly participated in religious activities, communicated more with others, received more social support, and realized the meaning of life from religious teachings.

We can implement targeted measures to enhance the meaning of life by identifying MIL sources in older adults. For life security, governments can further implement social welfare security policies. Moreover, increasing the intensity of medical insurance reimbursement, simplifying the process of medical treatment and reimbursement, enhancing material security, and improving the sense of gain and security can improve MIL in older adults. To improve family and social support, family members should strengthen communication with older adults, get along well with them, provide emotional and material support, and encourage them to participate in social activities and communicate with others. In addition, volunteers from the government and social organizations could accompany older adults to help them experience love and care. To improve a sense of value, corresponding posts can be provided according to their health to reflect their value. For example, encourage older adults to participate in volunteer activities, where the volunteer time can be deposited into a “time bank” for them to withdraw volunteer services when they need them. Providing equal employment opportunities for older adults and tapping their productivity can relieve social pressure and allow them to be active. They could also be encouraged to participate in community or nursing home construction by caring for flowers, plants, and vegetables to enhance their sense of value and meaning. The lives of older adults could also be improved by providing leisure activities, entertainment facilities, and physical exercise facilities in communities or parks, and encouraging them to attend parks and other beautiful and pleasant places to exercise, enrich their leisure life, and reduce the impact of loneliness, depression, anxiety, and other negative emotions, so that they can enjoy their old age healthily and happily. To enhance personal development, community education should be strengthened and corresponding learning classes should be established according to older adults’ needs. Local governments should attach importance to the education of older adults and focus on solving issues regarding fewer universities and enrollment. We should strengthen the construction of universities and community training institutions for older adults, and provide equipment and financial support. We should also encourage high-quality, professional, caring, and enthusiastic teachers to join in providing education for older adults, and improve the welfare of teachers who provide education for older adults. Moreover, we should provide rich educational content for them and add new technologies, such as health regimens, healthcare, computer, finance, financial management, mobile phone use, and traffic safety. We should broaden education channels for them, adopt modern educational means, such as multimedia and networks, and rely on “Internet Plus” to allow more older adults to enjoy education.

Our study has limitations. The convenience sampling method may limit the representativeness of this study’s sample. Future studies could use a multi-center stratified random sample to increase the representativeness and the universality of the research results. Moreover, we verified only one mediating variable, and the mediating effect of MIL accounts for only part of the total effect. Future research should explore other potential mediating variables related to MIL.

## 5. Conclusion

This study used the SMSE to measure sources and levels of MIL in Chinese older adults. Findings show that the level of MIL was high in older adults from Guangzhou, and influencing factors included depression, monthly income, personality, and family support. MIL partially mediated the relationship between family care and depression and completely mediated the relationship between family care and quality of life. Depression has a significant negative impact on the quality of life. Many factors influence MIL in older adults; we can improve their sense of meaning by improving their life security, increasing their sense of gain, promoting family care and social support, and identifying sources of meaning, to promote successful aging in the future.

## Data availability statement

The raw data supporting the conclusions of this article will be made available by the authors, without undue reservation.

## Author contributions

J-JZ conducted the research design, statistical analyses, and manuscript writing and revision. YZ conducted the data collection and manuscript writing and revision. Q-ZR and TL helped with the research design and data collection. G-DL, M-YL, S-HC, and PT helped with participants’ recruitment and data collection. Y-LG conceptualized the aims and hypotheses, participated in the overall design, and revised the manuscript. All authors gave critical comments, read and approved the final manuscript.

## Funding

This study was supported by the project of the Educational Commission of Guangdong Province of China (C1031707); the Sanming Project of Medicine in Shenzhen (SZZYSM202108013); and the Community Education Project of the Guangzhou community (2019SQJY010).

## Conflict of interest

The authors declare that the research was conducted in the absence of any commercial or financial relationships that could be construed as a potential conflict of interest.

## Publisher’s note

All claims expressed in this article are solely those of the authors and do not necessarily represent those of their affiliated organizations, or those of the publisher, the editors and the reviewers. Any product that may be evaluated in this article, or claim that may be made by its manufacturer, is not guaranteed or endorsed by the publisher.

## Abbreviations

SMSE: Sources of Meaning in life Scale for the elderly
APGAR: Family Care Index
CES-D-10: Center for Epidemiological Studies Depression Scale-10
EQ-5D: EuroqOL-5 Dimensions
MIL: Meaning in Life
QoL: Quality of Life
